# Expression of hypoxia‐inducible factor 1α, glucose transporter 1, and hexokinase 2 in primary central nervous system lymphoma and the correlation with the biological behaviors

**DOI:** 10.1002/brb3.1718

**Published:** 2020-06-12

**Authors:** Na Shen, Yaming Wang, Xuefei Sun, Xueyan Bai, Jinglan He, Qu Cui, Jun Qian, Hong Zhu, Yuedan Chen, Ruixian Xing, Qing Liu, Yuchen Wu, Junhong Li, Wenyuan Lai, Shengjun Sun, Nan Ji, Yuanbo Liu

**Affiliations:** ^1^ Department of Hematology Beijing Tiantan Hospital Capital Medical University Beijing China; ^2^ Department of Neurosurgery Xuanwu Hospital Capital Medical University Beijing China; ^3^ Department of Orthopedics Affiliated Hospital of Hebei University of Engineering Handan China; ^4^ Neuroimaging Center Beijing Tiantan Hospital Capital Medical University Beijing China; ^5^ Department of Neurosurgery Beijing Tiantan Hospital Capital Medical University Beijing China

**Keywords:** glucose transporter 1, hexokinase 2, hypoxia‐inducible factor 1α, primary central nervous system lymphoma, prognostic factors

## Abstract

**Background:**

It has been indicated that abnormal glucose metabolism mediated by hypoxia‐inducible factor 1α (HIF‐1α) played an essential role in the development of solid tumor. However, there were rare studies about the role of them in primary central nervous system lymphoma (PCNSL).

**Objective:**

To investigate the protein levels of HIF‐1α, glucose transporter 1 (GLUT1), and hexokinase 2 (HK2) in PCNSL and whether their levels are associated with prognostic factors.

**Methods:**

Expression of HIF‐1α, GLUT1, and HK2 in 39 tumor tissues was evaluated by immunohistochemical stainning. The correlation of the expression of HIF‐1α with the protein level of GLUT1 and HK2 was investigated. In addition, the association between these protein expression levels and clinical parameters and prognosis was analyzed.

**Results:**

In the tumor specimens of PCNSL, positive stainings of HIF‐1α, GLUT1, and HK2 were in 23 patients (58.97%), 25 patients (64.1%), and 26 patients (66.67%), respectively, which were associated with the expression level of lactic dehydrogenase (LDH), but not with age, gender, number of lesion, ECOG score, or deep structure. The expression of HIF‐1α was positively correlated with the expression of GLUT1 (*p* < .01, *r* = .749) and HK2 (*p* < .01, *r* = .787). Univariate analysis showed that upregulated GLUT1 was unfavorable predictors of progression‐free survival (PFS) in PCNSL. The results of Cox proportional hazards model showed GLUT1 was significantly associated with shorter PFS (hazard ration: 5.65; 95% confidence interval: 1.23–25.84; *p* = .026).

**Conclusions:**

This study indicated that there was a hypoxic microenvironment and HIF‐1α was involved in the regulation of glycolysis pathway in PCNSL. GLUT1 might be a potential marker for shorter PFS in PCNSL.

## Graphical abstract

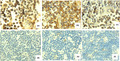

## 1 INTRODUCTION

Primary central nervous system lymphoma (PCNSL) is classified as a rare and aggressive form of malignant lymphoma, which is mainly confined to the brain, spinal cord, eyes, and leptomeninges without the presence of systemic lymphoma, accounting for <3% of primary intracranial tumors (Batchelor & Loefflfler, 2006). Approximately 95% of PCNSL are diffuse large B‐cell lymphoma (DLBCL). Despite the high‐dose methotrexate and/or rituximab‐based chemotherapy regimen, the therapeutic efficacy of PCNSL patients has been significantly improved. However, overall and long‐term survival remains challenging, and the five‐year survival rate was <30% (Shiels et al., 2016). Tumorigenesis of PCNSL is a complex processes involving various gene and mechanism. Lots of researches were carried out to demonstrate potential pathogenesis and associated genes and proteins, which aimed to improve diagnostic and treatment methods of PCNSL metabolic rearrangement, especially shifts in glucose metabolism, is a hallmark of tumors. Cancer cells have been shown to preferentially metabolize glucose to lactate even in the presence of oxygen, defined as the “Warburg effect” or aerobic glycolysis, which provides sufficient amounts of metabolic intermediates for anabolic processes of cancer cells (Lunt and Vander Heiden, 2011), and is required for sustaining tumor cell proliferation, aggressiveness, resistance to hypoxia, and apoptosis.

Hypoxia‐inducible factor 1α (HIF‐1α) is not only a critical transcriptional regulator but also a factor for cellular adaptation to hypoxic conditions. Under normoxic conditions, HIF‐1α, which is short‐lived, hydroxylated and then immediately ubiquitinated by E3 ubiquitin ligase before finally being degraded through the 26S proteasome. However, under the hypoxic microenviroment of tumor, HIF‐1α subunits are stabilized by coactivator proteins, which enhance them interaction with its binding partner HIF‐1β subunit. Subsequently, the product induces expression of many hypoxia‐responsive genes by binding to the hypoxia‐responsive enhancer sequence, the hypoxia‐response element (HRE) (Maxwell, Pugh, & Ratcliffe, 2001; Semenza, 2007). Furthermore, HIF‐1α activation leads to upregulation of glucose transporter 1 (GLUT1), glycolytic enzymes including hexokinase 2 (HK2), pyruvate dehydrogenase, and lactate dehydrogenase. Finally, the aerobic glycolysis is enhanced, but the oxidative phosphorylation pathway is inhibited (Kim, 2006; Semenza, 2011; Simon, 2006). Studies have demonstrated that HIF‐1α was activated under condition of hypoxia in DLBCL cells, which induced the expression of HK2 and GLUT1 (Bhalla et al., 2018).

Amounts of researches have been performed to identify the role of the Warburg effect in tumorigenesis of solid tumors and the correlation with poor prognosis, such as gastric cancer (Hao et al., 2019) and ovarian adenocarcinomas (Yasuda et al., 2008). However, there were few studies about the Warburg effect in patients with PCNSL. Our study aimed to investigate the expression of HIF‐1α, GLUT1, and HK2 in patients with PCNSL and evaluate their correlation with clinical parameters, further to provide potential methods of diagnosis and treatment for PCNSL.

## 2 MATERIALS AND METHODS

### 2.1 Patients

In this retrospective study, clinical data and tumor specimens of 39 patients diagnosed as PCNSL were collected in the department of hematology of Beijing Tiantan Hospital from January 2015 to December 2016. Diagnosis of DLBCL for all specimens was carried out by histologic review based on the Revised European‐American Lymphoma and WHO classification (Harris et al., 1994). All patients had received the regimen based on HD‐MTX. This study was approved by the Beijing TianTan Hospital Ethics Committee, Capital Medical University, and written informed consents were obtained from all patients.

### 2.2 Specimens

Tumor specimens were obtained by stereotactic biopsy or surgery. A total of 39 formalin‐fixed paraffin‐embedded tumor specimens originated from patients with PCNSL, and 10 paraffin‐embedded normal lymph node tissues originated from nontumor patients. These tissues were applied in the test of immunhistochemistry (IHC).

### 2.3 Immunohistochemical analysis

Serial sections (4 μm) were cut from tumor tissues and normal lymph node tissues for IHC staining. First, sections were dewaxed with xylene and dehydrated with alcohol. Second, sections were immersed in a citric acid buffer, and then, the antigens were extracted by microwave for 15 min. Third, sections were neutralized with endogenous peroxidase by 3% H_2_O_2_ for 15 min at room temperature and then were blocked by 5% goat serum for 1 hr. Fourth, sections were incubated with following primary antibodies: rabbit antihuman HIF‐1α monoclonal antibody (1:200), rabbit antihuman GLUT1 monoclonal antibody(1:1,000), rabbit antihuman HK2 monoclonal antibody(1:500). All the primary antibodies were incubated overnight at 4°C. Subsequently, these sections were incubated with HRP‐labeled secondary antibodies (1:200) at room temperature for 1 hr. Finally, the sections were incubated with DAB solution for 15 min at room temperature.

### 2.4 Scores of immunohistochemical staining

Two pathologists assessed the immunohistochemical staining independently using Zeiss light microscope. According to the intensity and degree of staining, the immunohistochemical results were scored semiquantitatively. Scoring of tumor cell staining is as follows: 0 is for negative, 1 is for light brown, 2 is for brown, and 3 is for dark brown. A total of 5 regions were simultaneously randomly selected with 400× magnification, and the proportion of positive cells in total fields of view was counted. The scoring of average positive cell rate of 5 fields is as follows: 1 is for 0%–25%, 2 is for 26%‐50%, 3 is for 51%‐75%, and 4 is for >75%. The total score is product of score of tumor cell staining and positive cell rate, and its value more than 3 can be considered to be positive.

For the evaluation of HIF‐1α, there was a different standard of grading. Immunoreactivity was categorized into following five semiquantitative classes based on the percentage of stained cancer cells: 0 (negative), 1 (1%–10% positive cells), 2 (11%–50% positive cells), 3 (50%–75% positive cells), and 4 (>75% positive cells). The scoring of immunostaining intensity determined by light microscopy is as follows: 0 is for negative, 1 is for weakly positive, 2 is for moderately positive, and 3 is for strongly positive. Finally, the total score is product of score of immunoreactivity and immunostaining intensity, and its value more than 3 can be considered to be positive.

### 2.5 Analysis of association between clinicopathologic variables and expression of HIF‐1α, GLUT1, and HK2 in PCNSL

The association between expression of HIF‐1α, GLUT1, and HK2 and clinicopathologic variables was analyzed, including age, gender, number of lesion, level of LDH, ECOG performance status, and deep structure involvement, which aimed to explore whether their protein levels can be used as a predictor of pathological state in PCNSL.

### 2.6 Analysis of correlation of HIF‐1α with GLUT1 and HK2 and their prognostic value in PCNSL

The analysis of correlation was carried out to investigate whether the expression of HIF‐1α was correlated with expression of GLUT1 and HK2 in our study. The curves of overall survival (OS) and progression‐free survival (PFS) were generated based on the Kaplan–Meier method, and the differences were analyzed by log‐rank test. Meanwhile, whether the upregulation of HIF‐1α, GLUT1, and HK2 were risk factors for OS and PFS in patients with PCNSL was analyzed in the univariate analysis. Furthermore, Cox proportional hazards model was established to evaluate prognostic value of expression of HIF‐1α, GLUT1, and HK2.

### 2.7 Statistical analysis

All data collected in our study were processed and analyzed by SPSS 22.0 software. The differences in HIF‐1α, GLUT1, and HK2 between tumor specimens and normal lymph node specimens were assessed by the chi‐squared (*χ*)^2^ test. The relationships between the expression of HIF‐1α, GLUT1, and HK2 and the clinicopathologic variables were evaluated by the Fisher's exact test and chi‐squared test. Spearman's rank correlation was used to test the correlation of continuous variables. Log‐rank test was used for univariate analysis, and Kaplan–Meier survival curve was constructed. Cox regression model was used for multivariate analysis. *p *< .05 was considered statistically significant.

## 3 RESULTS

### 3.1 Patient characteristics

All cases were confirmed as DLBCL, and the average age of the patients was 57 years old (ranged 30–82 years old), and the ratio of male to female was 17:22. There were 22 cases (56.41%) with LDH increased, 14 cases (35.90%) with multiple brain injuries, 27 cases (69.23%) with ECOG score >2, and 26 cases (66.67%) with deep brain involvement.

### 3.2 Immunohistochemical detection and association between expression of HIF‐1α, GLUT1, and HK2 and clinicopathologic variables in PCNSL

**FIGURE 1:**
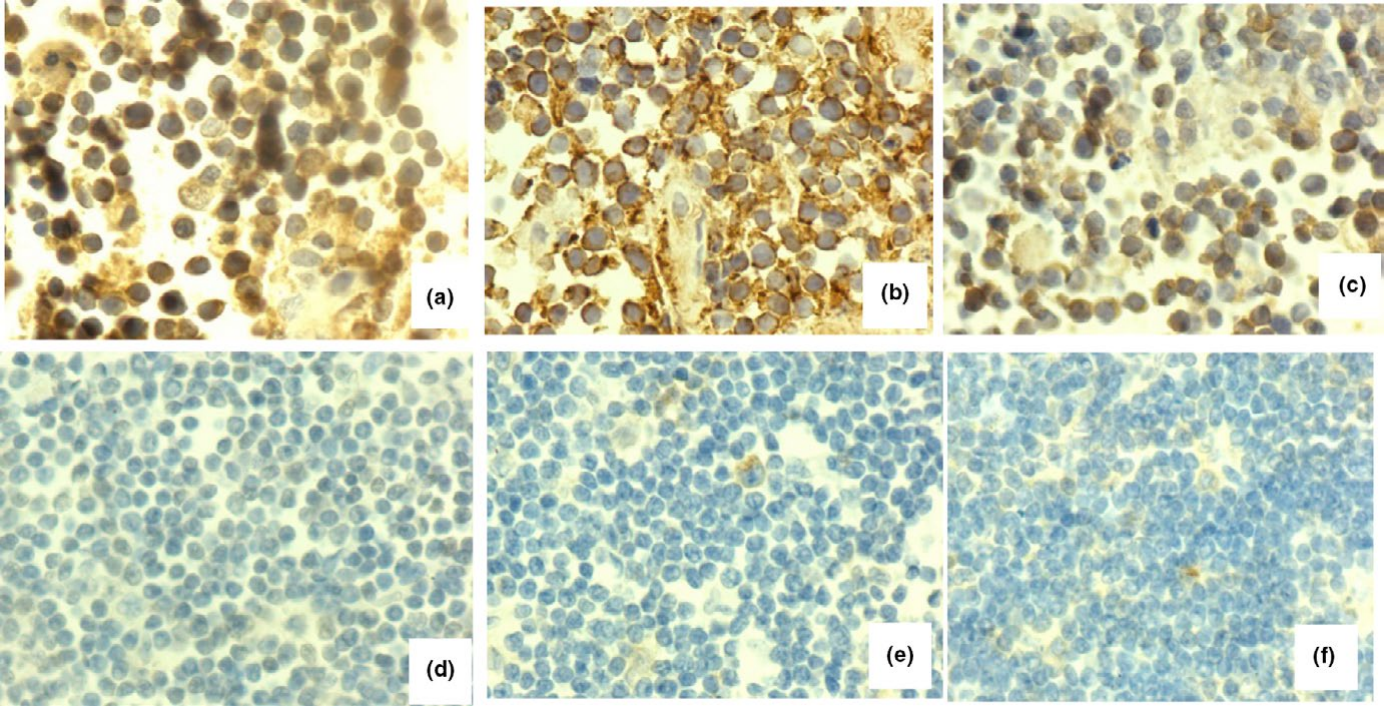
Immunohistochemical staining for hypoxia‐inducible factor 1α (HIF‐1α), glucose transporter 1 (GLUT1), and hexokinase 2 (HK2). (a‐c) A primary central nervous system lymphoma sample that was positive for HIF‐1α, GLUT1, and HK2. (d–f) A normal lymph node sample that was negative for HIF‐1α, GLUT1, and HK2

As described above, the expression of HIF‐1α, GLUT1, and HK2 was detected by semiquantitative analysis using immunohistochemical staining. HIF‐1α, GLUT1, and HK2 were stained and demonstrated in tumor cells (Figure 1). According to the criteria that tumors with scores of 4–12 were defined as positive for expression, our study showed that the expression rates of HIF‐1α, GLUT1, and HK2 were 58.97% (23/39), 64.10% (25/39), and 66.67% (26/39), respectively, suggesting that HIF‐1α, GLUT1, and HK2 were significantly upregulated in tumors compared with the normal lymph node tissue (*p* < .01; Table 1). Meanwhile, our results found that the expression of HIF‐1α was particularly associated with the level of LDH (*p* < .05), but not with age, gender, number of lesion, ECOG score, or deep structure (*p *> .05). In addition, similar results were found in terms of GLUT1 and HK2, which were also associated with the expression level of LDH (*p *< .05), but not with age, gender, number of lesion, ECOG score, or deep structure (*p *> .05; Table 2).

**TABLE 1:** HIF‐1α, GLUT1, and HK2 expression in normal lymphoma tissues and PCNSL tissues

Specimen	*n*	HIF‐1α			HK2			GLUT1		
		+	−		+	−		+	−	
PCNSL tissue	39	23	16	*χ* ^2^ * = 8.873*	26	13	*χ* ^2^ * = 11.652*	25	14	*χ* ^2^ * = 10.648*
Normal lymphoma tissues	10	0	10	*p* < .01	0	10	*p* < .01	0	10	*p* < .01

**TABLE 2:** Clinical characteristics of patients in PCNSL in relation to HIF‐1α, GLUT1, and HK2 protein expression

Variables	HIF protein expression (*n* = 39)			HK2 protein expression (*n* = 39)			GLUT1 protein expression (*n* = 39)		
	+ (*n* = 23)	− (*n* = 16)	*p*	+ (*n* = 26)	− (*n* = 13)	*p*	+ (*n* = 25)	− (*n* = 14)	*p*
Age (years)									
>60 (*n* = 13)	8	5	>.05	9	4	>.05	8	5	>.05
≤60 (*n* = 26)	15	11		17	9		17	9	
Gender									
Male (*n* = 17)	12	5	>.05	12	5	>.05	13	4	>.05
Female (*n* = 22)	11	11		14	8		12	10	
LDH									
Elevated (*n* = 22)	17	5	<.05	19	3	<.01	18	4	<.05
Normal (*n* = 17)	6	11		7	10		7	10	
No. of lesion									
1 (*n* = 25)	15	10	>.05	18	7	>.05	15	10	>.05
At least 2 (*n* = 14)	8	6		8	6		10	4	
ECOG performance status									
0–1 (*n* = 12)	5	7	>.05	6	6	>.05	5	7	>.05
At least 2 (*n* = 27)	18	9		20	7		20	7	
Deep structure involvement									
Presence (*n* = 26)	17	9	>.05	18	8	>.05	17	9	>.05
Absence (*n* = 13)	6	7		8	5		8	5	

### 3.3 Correlation of HIF‐1α expression with GLUT1 and HK2

Whether the HIF‐1α was associated with GLUT1 and HK2 were also analyzed in our study. There was a positive correlation between HIF‐1α and GLUT1 expressions (*p *< .01, *r* = .749; Table 3). In addition, the expression of HIF‐1α was particularly correlated with expression of HK2 (*p* < .01, *r* = .787; Table 3).

**TABLE 3:** Relationship between the expression of HIF‐1α and the expression of GLUT1 and HK2 in PCNSL

Marker	*n*	HIF−1α			Spearman's rank correlation	
		+ (*n* = 23)	− (*n* = 16)	Positive ration (%)	*r*	*p*
HK2 (+)	26	21	5	80.76	.787	<.01
HK2 (−)	13	2	11	15.38		
GLUT1 (+)	25	21	4	84.0	.749	<.01
GLUT1 (−)	14	2	12	14.29		

### 3.4 Prognostic value of HIF‐1α, GLUT1, and HK2 expression in PCNSL

The median follow‐up was 31.5 months (ranged 11–44 months). At the end of follow‐up, 13 patients died and 26 survived. The 3‐year OS and PFS rates were 51.6% and 38.5%, respectively. The median PFS time was 26 months (95% confidence interval: 19.25–32.75), but the median OS time was not reached.

**FIGURE 2:**
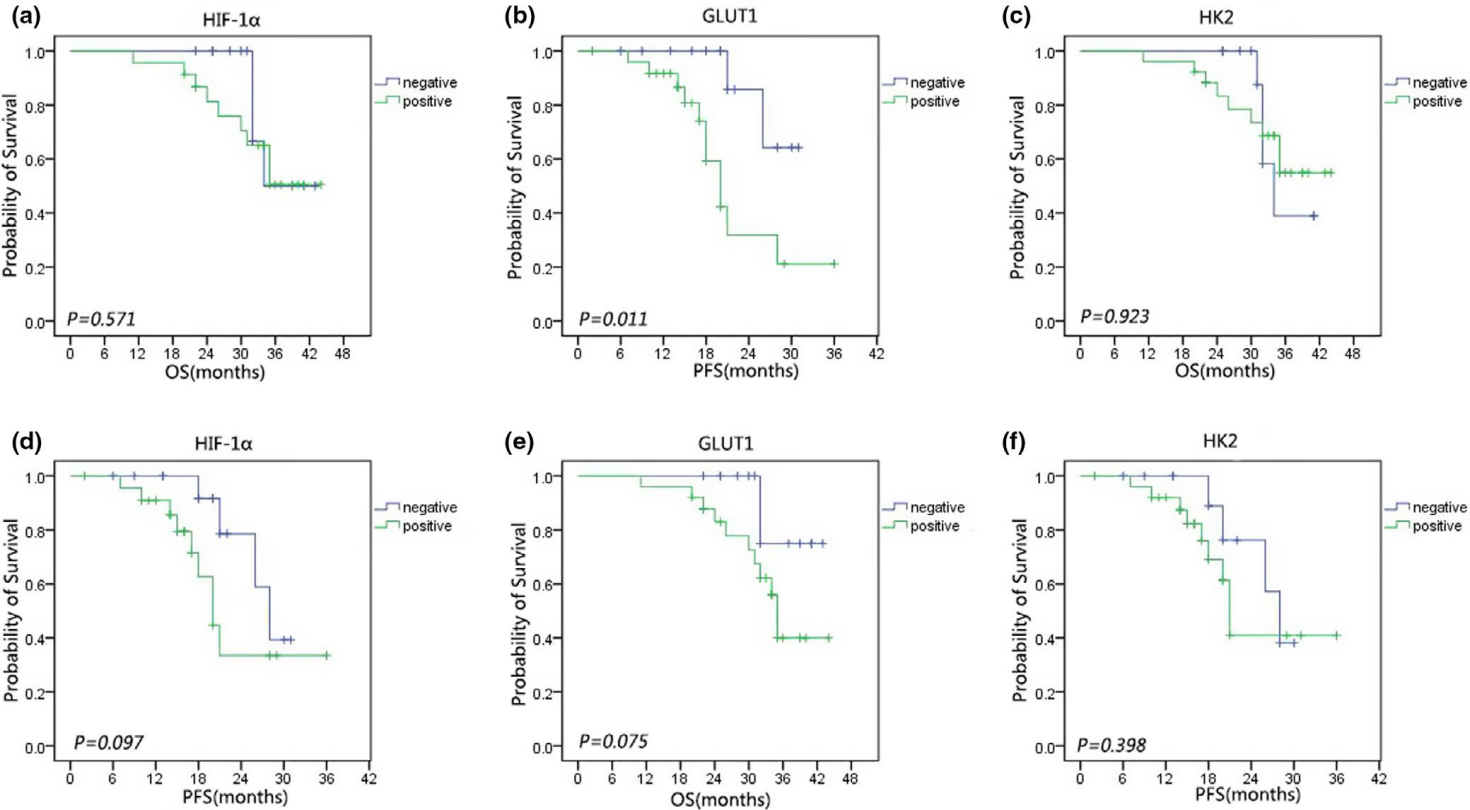
Kaplan–Meier curves of overall survival (OS) rates of primary central nervous system lymphoma for (a) patients with positive and negative HIF‐1α immunostaining, (b) patients with positive and negative GLUT1 immunostaining, and (c) patients with positive and negative HK2 immunostaining. Kaplan–Meier curves of progression‐free survival (PFS) rates of primary central nervous system lymphoma for (d) patients with positive and negative HIF‐1α immunostaining, (e) patients with positive and negative GLUT1 immunostaining, and (f) patients with positive and negative HK2 immunostaining

Univariate analysis demonstrated that upregulation of GLUT1 was risk factor for PFS in patients with PCNSL (*p* = .011) (Figure 2b), but not for OS (*p* = .075) (Figure 2e). However, overexpression of HIF‐1α and HK2 had no significant effect on OS and PFS in patients with PCNSL (*p* = .571, *p* = .097, *p* = .923, and *p* = .398, respectively) (Figure 2a,c,d,f). These results suggested that GLUT1 might be considered as an important prognostic marker in patients with PCNSL. Using the Cox proportional hazards model, the results showed that GLUT1 was significantly associated with shorter PFS (hazard ratio: 5.65; 95% confidence interval: 1.23–25.84; *p* = .026). However, no independent prognostic factors have been found.

## 4 DISCUSSION

It is well known that under conditions of hypoxia, HIF‐1α plays an essential role in the transcription of various genes involved in physiological reaction, such as angiogenesis, tumor growth, invasion, metastasis, and glucose metabolism (Kim, Park, Lee, & Kim, 2017; Leung et al., 2017; Soni & Padwad, 2017). It has been reported that overexpression of HIF‐1α caused treatment failure and a poor prognosis of brain tumors (Zagzag et al., 2000). HIF‐1α was upregulated to accommodate for hypoxic conditions, and its upregulation may be one of the factors inducing glycolysis under hypoxia (Hanahan & Weinberg, 2011).

In this study, immunohistochemistry showed that the positive rate of HIF‐1α protein was 58.97% in tumors, which was significantly higher than that in the normal lymph node tissue. HIF‐1α was synthesized in cytoplasm and transferred to the nucleus, and then combined with HIF‐1β for activation; thus, cytoplasmic and nuclear staining can be exhibited in positive cells (Lee et al., 2009). It has been reported that the protein level of HIF‐1α in the patients with myelodysplastic syndrome (MDS) upregulated compared with the control group, which was also found to be associated with poor prognosis (Liang et al., 2019). It is well known that HIF‐1α plays an essential role in the tumorigenesis of solid tumor. However, Kaplan–Meier survival analysis demonstrated that PCNSL patients with positive expression of HIF‐1α had no correlation with lower OS and PFS in this study, which was consistent with Jun A. KIM's reports (Kim et al., 2011).

Rapidly multiplying tumor cells tend to consume large amounts of glucose to provide energy for their proliferation and development (Courtnay et al., 2015). Under condition of hypoxia, HIF‐1α directly regulates the expression of genes related to glycolysis and simulates glucose consumption. Upregulated GLUT1 induced by HIF‐1α has been reported to increase cellular uptake of glucose and enhance aerobic glycolysis in tumor cells. Although expression of GLUT1 in normal tissues is restricted, higher levels have been found in erythrocytes, brain, cartilage, and retinal and placental tissue. However, GLUT1 protein is widely overexpressed in lots of human malignancies, including nonsmall‐cell lung cancer, colorectal cancer, breast cancer, and gastric cancer (Macheda, Rogers, & Best, 2005). In this study, our results demonstrated that the positive rate of GLUT1 was 64.1% in patients with PCNSL, which exhibited significant difference compared with normal lymph node tissue. It has been reported that metabolic rearrangement of tumors was also regulated by the activation of proto‐oncogenes (c‐Myc), transcription factor (HIF‐1α), signaling pathways (PI3K/AKT signal pathway), and the inactivation of tumor‐suppressor genes (p53), in addition to the requirement of energy (Hamanaka and Chandel, 2012). Furthermore, the test of Spearman's rank correlation found that there was positive correlation between the protein expression of HIF‐1α and GLUT1, which was consistent with previous study (Chen et al., 2018). Besides, these results also suggested that upregulated GLUT1 was positively correlated with lower PFS, not with lower OS.

HK2, the first enzyme in the glycolytic pathway catalyzing the reaction in which glucose is phosphorylated into glucose‐6‐phosphate, has been identified overexpressed in numerous tumors, such as laryngeal carcinoma (Chen, Zhang, Li, Tang, & Kong, 2014) and breast cancer (Marini et al., 2013). Studies have shown that overexpression of HK2 not only enhanced the rate of glycolysis, but also was necessary for tumorigenesis (Patra et al., 2013). Mitochondria‐associated HK2 protects cells from mitochondrial death pathways by restricting mitochondrial permeability transition pores (mPTPs). In this study, positive expression of HK2 was identified in 66.67% of patients with PCNSL, which exhibited significant difference compared with normal lymph node tissue. It was found that level of HK2 was higher in DLBCL than that in non‐DLBCL (Shim et al., 2009). In non‐Hodgkin's lymphoma (NHL), a series of anomalies lead to aberrant activation of the PI3K/Akt/mTOR pathway (Blachly & Baiocchi, 2014). Phosphorylation of Akt suggests the activation of PI3K signaling pathway, inducing the expression of HIF‐1α, further rendering tumors dependent on glycolysis for survival by increasing the expression of GLUT1 and glycolytic enzymes, such as HK2 (Fan, Dickman, & Zong, 2010; Inoki, Corraadetti, & Guan, 2005; Robey & Hay, 2009). Furthermore, our data demonstrated that there was significant correlation between HIF‐1α and HK2. It has been demonstrated that there was no correlation between HK expression and prognosis, and no significant difference was found in OS and PFS between HK2‐positive and HK2‐negative cases.

There are also some limitations in our study. Our sample size was small, and the sample size needs to be expanded for further confirmation. Because PCNSL lacks corresponding cell lines, further verification tests in vitro are difficult to conduct. More parameters related to prognosis evaluation should be investigated in further study.

In conclusion, this study indicated that the protein levels of HIF‐1α, GLUT1, and HK2 were significantly higher in PCNSL than that in normal lymph node. In addition, there was positive correlation between the expression of HIF‐1α and the expression of GLUT1 and HK2. PCNSL patients with positive expression of GLUT1 had a lower PFS. However, there was no independent predictive factor for poor prognosis in patients with PCNSL. Therefore, there was a hypoxic microenvironment and HIF‐1α was involved in the regulation of glycolysis pathway in PCNSL. GLUT1 might be a potential marker for shorter PFS in PCNSL. Our results may contribute to understand the effect of abnormal glucose metabolism mediated by HIF‐1α on prognosis. However, these conclusions need to be further verified by expanded samples and in vitro experiments.

## CONFLICTS OF INTEREST

There is no conflict of interest.

## AUTHOR CONTRIBUTIONS

Na Shen and Yuanbo Liu contributed to the conception and design of the study. All authors participated in the clinical practice, including diagnosis, treatment, consultation, and follow‐up of patients. Na Shen and Yaming Wang contributed to the acquisition of data. Xuefei Sun and Xueyan Bai contributed to the analysis of data. Na Shen wrote the manuscript. Yuanbo Liu revised the manuscript. All authors approved the final version of the manuscript.

## Data Availability

The datasets generated and analyzed during the current study are available from the corresponding author on reasonable request.
